# Effects of Age at First Joining and Ewe Genotype on the Performance of Two-Tooth Ewes and That of Their Progeny to Slaughter

**DOI:** 10.3390/ani12050653

**Published:** 2022-03-04

**Authors:** Timothy W. J. Keady, James P. Hanrahan

**Affiliations:** 1 Teagasc, Animal & Grassland Research & Innovation Centre, Mellows Campus, Athenry, H65 R718 Co. Galway, Ireland; 2 School of Veterinary Medicine, University College Dublin, D04 V1W8 Dublin, Ireland; sjohanrahan@gmail.com

**Keywords:** grazing, Suffolk, Belclare, litter size, body condition score, body size, mortality, lamb, carcass weight

## Abstract

**Simple Summary:**

Ewe replacements are a major cost in prime-lamb production. Many producers do not join replacements at 7 months as they believe that it has a negative effect on two-tooth performance. The effect of age at first joining on the reproductive performance of two-tooth ewes, representing three genotypes, was evaluated as well as the performance of their progeny. Whilst joining at 7 months reduced ewe body weight when joined at 19 months and immediately post joining, body weight at the subsequent lambing was increased, and ewe productivity and progeny performance were unaffected. Ewe genotype significantly influenced litter size and the number of lambs reared per ewe joined but had no effect on progeny performance. The probability of rearing at least one lamb (PR1L) is an indicator of overall efficiency. In the current study, whilst there was no relationship between ewe body weight when joined at 19 months and PR1L, there was a positive relationship between body weight at 7 months and PR1L at 2 years. It is concluded that joining replacements at 7 months does not have a negative impact on the performance of two-tooth ewes.

**Abstract:**

The effects of first-joining age (7 or 19 months) and genotype on ewe performance when joined to lamb at 2 years, and the performance of their progeny, were evaluated using 424 ewes, representing 3 genotypes: Belclare (Bel), Suffolk × Belclare (Suf × Bel) and Suffolk-type (≥75% Suffolk ancestry (Suf75)). Ewes were managed in a grass-based system. Ewes first joined at 7 months were lighter (*p* < 0.01) at 19 months and immediately post lambing; otherwise, age at first joining had no effects (*p* > 0.05) on ewes or their progeny and there were no important interactions with genotype. Bel and Suf × Bel had larger litters (*p* < 0.001) and reared more lambs per ewe joined (*p* < 0.01) than Suf75. Ewe genotype had no effect (*p* > 0.05) on proportion failing to lamb, incidence of lambing assistance, lamb mortality, ewe survival to 31 months, or progeny performance. Increasing the body weight of ewes at 7 months of age increased the probability (*p* < 0.02) of rearing 1 or more lambs at 2 years and there was no interaction with genotype. It is concluded that age at first joining had no negative impact on the performance of ewes or their progeny.

## 1. Introduction

Replacement ewes represent a major cost in prime-lamb production and Keady [1] reported that the mean cost, nationally, of producing a replacement ewe, when joined for the first time at ~19 months, equated to approximately 25% of the value of her lifetime lamb-carcass output. Since the costs involved in sheepmeat production are attributable primarily to the ewe, rather than to her lambs, production costs per lamb can be reduced by increasing the number of lambs produced per ewe lifetime. Two major determinants of the number of lambs reared per ewe lifetime are prolificacy and the number of lamb crops produced. Ewe genotype is a major determinant of prolificacy while age at first joining may affect the number of lamb crops per ewe lifetime. We have reported [2] that the productivity of replacements joined to lamb at 1 year depends on genotype: Belclare ewes reared 1.17 lambs per ewe joined compared with 0.82 for Suffolk-cross ewes of the type prevalent in lowland flocks in Ireland. Furthermore, in that study, all lambs were slaughtered prior to the end of the grazing season (mean carcass weight of 20.6 kg). Many producers do not join their replacement ewes as ewe lambs because of a fear, or perception, of having a negative effect on subsequent growth/development and, consequently, on productivity. Kenyon et al. [3] concluded that the main reason farmers in New Zealand do not join replacements to lamb as 1-year-olds are the perceived negative effects on two-tooth performance. Previous authors have reported that, when lambing at 2 years of age, ewes that had previously lambed at 1 year were lighter at joining [4,5] and gave birth to fewer lambs [4,5]. However, Vavra et al. [6] reported that age at first lambing had no effect on the number of lambs born to 2-year-olds but the number reared was higher for those that had lambed at 1 year whilst McMillan and McDonald [7] reported that ewes producing their second litter at 2 years of age reared more lambs per ewe joined. Keady and Hanrahan [8,9] reported that altering the weight of two-tooth ewes at joining by 8.4 kg did not alter the number of lambs born or the number reared per ewe joined. Most previous studies of the effect of age at first lambing on the performance at 2 years involved ewes of relatively low productivity and which lambed outdoors [4,5]. There is little information on the effects of age at first lambing on performance of two-tooth ewes representing genotypes differing widely in prolificacy and, thus, level of productivity, and that are housed during mid and late pregnancy to facilitate higher stock carrying capacity in grass-based, prime-lamb systems. 

Many factors influence the efficiency of prime-lamb production within the farm gate. From a review of studies undertaken at the Athenry Research facility, it was concluded [10] that the number of lambs reared per ewe joined and system of grassland management are the two main determinants of the efficiency of prime-lamb production in grass-based systems. Ewe genotype is a key determinant of ewe productivity [9,10,11,12,13,14]. Ewe productivity has remained relatively static, at approximately 1.3 lambs weaned per ewe joined, on Irish lowland farms for the last 30 to 40 years [15,16,17]. This lack of an improvement is most probably attributable to the absence of significant increase in the use of more prolific ewe genotypes. Currently, 66% of lowland ewes in Ireland have been sired by one of the two main terminal sire breeds (Suffolk and Texel) [18], both of which have inherently low productivity [11]. The Belclare breed was developed from a range of genetic resources [19,20], has a litter size of approximately 2.2 under typical on-farm management conditions, and represents the sire of 9.5% of ewes in lowland flocks [18]. Belclare-cross ewes have a higher prolificacy than a wide selection of other crossbred types [11,14]. Thus, Hanrahan [11] reported that litter size and number of lambs reared per ewe joined were 0.20 and 0.21 higher for ewes having Belclare sires than crossbred ewes sired by the Suffolk breed whilst ewe lambs by Belclare sires reared 0.19 more lambs per ewe joined than Suffolk-sired contemporaries. 

In an earlier paper from this study [2], we reported that whilst replacements joined to lamb at 1 year of age, and which were offered a preferential plane of nutrition during mid and late pregnancy to meet requirements for pregnancy and growth, reared one lamb per ewe joined (difference among ewe genotypes was 0.35 lambs per ewe joined), BW at 19 months was reduced by 2 kg only. The hypotheses underlying the design of this study, which is part of an evaluation of the effect of joining to lamb at 1 year on lifetime performance, were that: (i) age at first joining (7 or 19 months) has no negative effects on ewe productivity when joined at 19 months; (ii) productivity of ewes joined at 19 months is influenced by genotype but that there is no interaction between genotype and any effect of age at first joining; (iii) lambs born to ewes joined at 19 months can yield suitable carcasses before the grazing season ends without the need for supplementation with concentrate (except for triplet-reared lambs during the pre-weaning phase).

## 2. Materials and Methods

### 2.1. Animals and Management

A total of 424 March-born two-tooth (~19 months old) ewes were used in this study, which represents the next phase in an investigation of the lifetime performance-effects of joining at 7 months [2] that was replicated (cohorts) over 2 consecutive years. All ewes remain on this study unless culled for normal husbandry/health reasons (mastitis, vaginal prolapse, foot defects or an unexpected decline in body condition), or they die. This study involves 3 genotypes that differ in expected prolificacy, and the animals initially assigned to this study were chosen to have average BW, at ~7 months, equal to 55% of estimated mature BW for their genotype. The details of the experimental design and initial numbers assigned to the 2 treatment groups (joined at ~7 months or not joined until ~19 months) may be found in [2]. For the initial phase of this study, the ewes joined at ~7 months were managed separately from the ‘not-joined’ group between joining and subsequent weaning, at which point they were combined and grazed on perennial-ryegrass (predominantly) swards. See [2] for full details of the grazing management and plane of nutrition offered from the time of joining at ~7 months of age up to joining at ~19 months of age.

For the present phase of this study, the number (number representing the 2 cohorts) of ewes available for each genotype were: 157 (67, 90) for Belclare (Bel); 114 (64, 50) for F1 Suffolk × Belclare (Suf × Bel; out of Bel ewes) and 153 (65, 88) for ewes with ≥75% Suffolk ancestry (Suf75; Suffolk rams by Suffolk-cross ewes). Oestrus was synchronized using progesterone-impregnated sponges, which were removed after 12 days; ewes were joined with Charollais rams (ewe:ram ratio 8:1) at 36 h post sponge removal. Rams were removed after 4 days and returned approximately 7 days later to cover any repeats. 

At the beginning of December, all ewes were housed in a slatted-floor shed (10–20 ewes per pen) where grass silage, sufficient to allow a refusal of 50 to 100 g/kg, was offered once daily. Water was available *ad libitum*. Ewes were shorn within 7 days of housing and expected litter size was ascertained by ultrasonography in mid-January. Following ultrasonography, ewes were re-penned according to expected litter size to facilitate differential concentrate supplementation during late pregnancy. A booster vaccine for pasturella pneumonia and clostridial disease (Heptavac-P, MSD Animal Health, Buckinghamshire, England) was administered ~4 weeks prior to lambing.

Ewes identified as carrying singles, twins, triplets or quads received total supplement of 14.5, 20.5, 27.5 and 30.5 kg concentrate, respectively, during late pregnancy in year 1; because the silage available in year 2 had a lower feed value, the corresponding concentrate amounts were 16, 23, 29 and 32 kg. The concentrate was offered to single-, twin-, triplet- and quadruplet-bearing ewes during the final 5, 6, 7, and 7 weeks of pregnancy, respectively; the initial amount per ewe was 0.3 kg/day in all cases. This amount was increased stepwise (by 0.1 or 0.2 kg, at 1 or 2 week intervals) to maxima that ranged from 0.6 kg (single-bearing ewes) to 1 kg (ewes with quads). Concentrate was offered once daily at ~10 AM. In year 1, the concentrate was formulated to have crude protein (CP) and metabolizable energy (ME) concentrations of 220 and 12.5 MJ/kg DM, respectively. The concentrate (pelleted) consisted of (g/kg fresh weight): barley (300), soybean hulls (175), soybean meal (160), rapeseed meal (90), maize (50), maize gluten (160), molasses (40) and minerals plus vitamins (25). The concentrate for year 2 was formulated to have CP and ME concentrations of 220 and 12.8 MJ/kg DM, respectively. The concentrate (pelleted) consisted of (g/kg fresh weight): barley (170), soybean hulls (145), soybean meal (200), rapeseed meal (80), maize (190), molassed sugar beet pulp (100), distillers grains (40), molasses (50) and minerals plus vitamins (25).

Ewes lambed indoors and were transferred to pasture, with their lambs, within 3 days of lambing. Those rearing singles or twins were managed as a single flock in a rotational-grazing system and received no concentrate supplement. Triplet-rearing ewes were managed in a separate flock and offered concentrate (0.5 kg/head; the same concentrate as offered during late pregnancy) daily until week 5 post lambing. Lambs being reared as triplets had access to concentrate (maximum 300 g/day per lamb) until weaning. Male lambs were not castrated and ewes that had 4 live lambs had at least 1 removed to an artificial rearing unit. Lambs were treated for internal parasites at 5 and 10 weeks of age (levamisole hydrochloride; Chanelle, Loughrea, Co. Galway, Ireland) and at 14 weeks (oral ivermectin (Oramec); Merial Animal Health, Harlow, Essex, England). Ewe and ram lambs were drafted at weaning if BW exceeded 43 and 44 kg, respectively; subsequent drafting was at approximately monthly intervals with BW criteria of 45 and 46 kg up to end of August, and 47 and 48 kg thereafter.

### 2.2. Forages

Herbage, the primary growth of permanent swards (mainly perennial ryegrass), was ensiled, following wilting (24 h), in mid-May each year, for the indoor-feeding period. The procedures employed were as previously described [21].

### 2.3. Measurements

Each year, the silages to be used were sampled (4 core samples per silo) ~1 week before ewes were housed; these samples were used for the determination of DM, CP, ammonia-N, DM digestibility (DMD) and ME concentrations as described in [22]. 

Body weight was recorded for all ewes pre joining (early October), post joining (late November), mid pregnancy (mid January), at lambing, 5 weeks post lambing, weaning (July) and pre joining, for the subsequent season, at 31 months. Ewe survival was defined for all ewes that were assigned to this study at 7 months (1 for ewes present for joining at 31 months, 0 otherwise). Ewe body condition score (BCS) [23] was recorded pre joining, post joining, at lambing, at approximately week 5 post lambing, and at weaning. Withers height, body length, chest (heart) girth and circumference of the cannon bone were recorded for all ewes post mating (early November), as described previously [8]. This collection of linear measurements will be referred to as body size. 

Where a ewe needed assistance with lambing, the level of assistance was recorded as described previously [9]. Lambs were tagged and weighed within 24 h of birth; lambs that were dead at this time were recorded as born dead. Total lamb mortality refers to those born dead plus lambs that died between birth and weaning. Lamb weights were recorded at approximately 5, 10 and 14 weeks of age and BW gain calculated for the intervals described previously [2]. Procedures relating to slaughter and carcass measurements were as described in [24]. 

### 2.4. Statistical Analyses

All data analyses involved the use of SAS/STAT software (Version 9.4 for Windows; 2002–2012); ewe traits were analyzed by fitting linear models using either Proc GLM (BW, BCS, body size, litter size, number of lambs reared) or Proc GENMOD with a logit link function (ewe survival, fertility, lambing assistance, lamb mortality and whether ≥1 lamb was reared). Proc MIXED was used for the analysis of lamb growth and carcass traits by fitting a linear mixed model with dam as a random effect. All models used had fixed effects for treatment, ewe genotype and season. Two-way interactions among these fixed effects were evaluated in initial models and any interaction with *p* ≤ 0.2 was retained in the final model; otherwise, the interaction terms were dropped. Fixed effects for sex, and birth type or birth-rearing category were added to models for analysis of lamb performance traits; since only 5 sets of triplets were reared (all to Bel ewes; 4 sets in year 1), these were included in the triplet-reared-as-twin category while triplets and twins reared as singles were combined. Artificially reared lambs were excluded from all analyses of lamb traits except for BW at birth and lamb mortality. Differences among genotypes were evaluated after adjustment for multiple comparisons (Tukey–Kramer). Analyses were also undertaken to evaluate the effects of ewe BW at 7 and 19 months on whether zero or ≥1 lamb was reared. In these analyses, the values for BW were deviations from the mean of appropriate genotype-x-treatment-x-season subclass.

## 3. Results

The means for pH, and concentrations of DM, ammonia N, CP, ME and DMD of the silage offered were: 4.0, 210 g/kg, 7.5 g/kg N, 106 g/kg DM, 11.2 MJ/kg DM and 730 g/kg DM, respectively, in year 1, and 4.2, 199 g/kg, 110 g/kg N, 117 g/kg DM, 10.7 MJ/kg DM and 700 g/kg DM, respectively, in year 2.

Least squares means by age at first joining and ewe genotype are presented in Table 1 for ewe BW, BCS and body size together with a summary of the statistical analysis of the effects of these factors. Ewes joined for the first time at 7 months were lighter and had a lower BCS pre joining (*p* < 0.001) and post joining (*p* < 0.01) but were heavier at lambing (*p* < 0.01) and 5 weeks post lambing (*p* < 0.05). There were, however, some significant interactions between these effects and year. In the case of BW up to and including in mid January, the interactions reflected the fact that, in year 1, ewes joined for the first time at 19 months were marginally lighter than those joined at 7 months, whereas, in year 2, those first joined at 19 months were up to 4 kg heavier (*p* < 0.01) than their contemporaries joined at 7 months. In contrast, for BW at lambing and 5 weeks post lambing, the pattern of differences was reversed so that, for the year 1 cohort, ewes joined at 7 months were heavier than those not joined until 19 months (*p* < 0.05), whereas the corresponding differences for the year 2 cohort were of opposite sign and not significant (*p* > 0.05). 

The interaction between ewe genotype and age at joining was significant (*p* < 0.05) for BW in mid January, at weaning and at 31 months, and for BCS at 5 weeks post lambing and at weaning. These effects reflected the varied ranking of genotypes between treatment groups. For BW, the BelxSuf was the heaviest at all time points for ewes joined for the first time at 19 months, whereas the heaviest genotype among those joined at 7 months was either Bel at joining or Suf75 at the other time points. However, the maximum difference among genotypes within treatment groups was ≤4.2 kg. The interactions for BCS reflected the observation that BCS was higher (*p* < 0.05) for Suf75 joined at 7 months than for Suf75 ewes joined first at 19 months. Ewes that had their first joining at 7 months had a larger body size (*p* < 0.01), essentially, reflecting the difference in body length. Relative to the BW of Bel ewes, that of Suf × Bel and Suf75 ewes was greater pre joining (*p* < 0.05 or smaller), post joining (*p* < 0.05 or smaller) and at lambing (*p* < 0.01). Ewe genotype was significant (*p* < 0.001) for body size post mating, which reflects the fact that Suf × Bel and Suf75 ewes had a lower withers height (*p* < 0.05), shorter body (*p* < 0.05) and greater cannon bone circumference (*p* < 0.05) than Bel ewes. Otherwise, there were no effects (*p* > 0.05) associated with age at first joining or ewe genotype for ewe BW, BCS or body size. Neither age at first joining nor ewe genotype had a significant effect on ewe survival to 31 months. 

The effects of age at first joining and ewe genotype on reproductive performance traits and lamb mortality are summarized in Table 2. Litter size for Suf75 was significantly lower (*p* < 0.01) than both Bel and Suf × Bel while the number of lambs reared per ewe joined was lower than for Bel (*p* < 0.01) and BelxSuf (*p* < 0.05). There was evidence for an interaction (*p* < 0.06) between age at joining and ewe genotype for failure to lamb, reflecting the exceptionally low incidence for BelxSuf ewes first joined at 19 months (see Table 2 footnote). Otherwise, neither first-joining age nor ewe genotype affected litter size, number reared, assistance at lambing or lamb mortality. 

**Figure 1:**
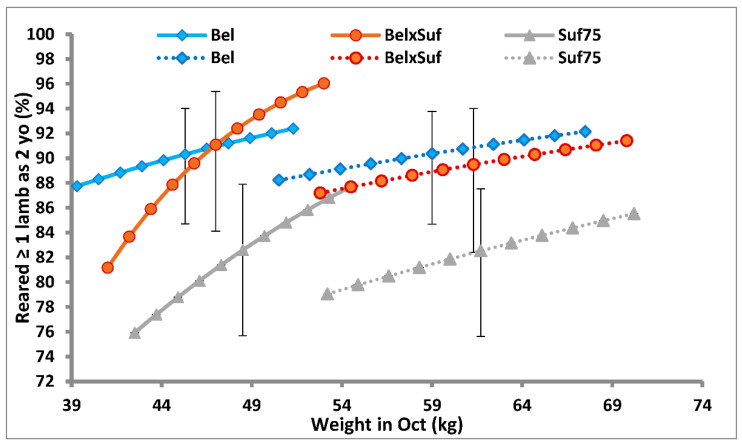
The effects of ewe genotype (Bel = Belclare, Bel × Suf = Belclare × Suffolk, Suf75 = ≥75% Suffolk ancestry) and body weight at 7 months of age (solid lines) and 19 months of age (broken lines) on the probably of rearing ≥ 1 lamb when lambing at 2 years of age (vertical bars show the 95% confidence interval at the mean BW for each genotype).

The relationships of BW at 7 and 19 months with the probability of rearing at least 1 lamb, when lambing at 2 years, are presented in Figure 1 for each genotype. As ewe weight at 7 months increased, the probability of rearing ≥1 lamb increased (*p* = 0.02) but the effect of BW at 19 months did not reach significance (*p* = 0.13); when both weights were included in the model, the effect of BW at 7 months was reduced slightly (*p* < 0.07), whereas that associated with BW at 19 months was near zero (*p* = 0.9). The correlation between BW at 7 and 19 months was 0.62. 

**Figure 2:**
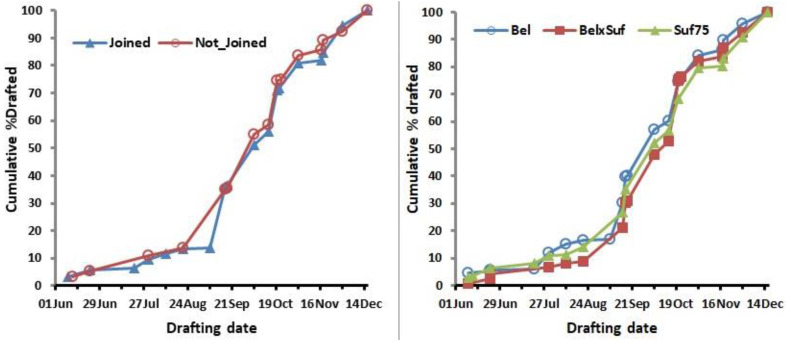
Cumulative drafting pattern (%) by treatment and genotype.

Effects on lamb growth and carcass traits are presented in Table 3 and the cumulative percent drafted by date is shown in Figure 2 by treatment and genotype. The interaction between age at first joining and ewe genotype was not significant (*p* > 0.05) for any of the lamb growth or carcass traits. There were, however, interactions (*p* < 0.05) between year and age at first joining for a number of growth traits; in all cases, this reflected the fact that in year 1, the performance of lambs born to ewes first joined at 7 months was better than those from contemporary ewes first joined at 19 months of age, whereas the reverse was the case for the second cohort. However, the differences between treatments involved were small relative to the trait means (of the order of 5% or less). Lambs born to ewes first joined at 7 months were significantly heavier at birth (*p* < 0.001) than those not joined at 7 months, and had a higher growth rate between birth and 5 weeks of age (*p* < 0.06), but did not differ (*p* > 0.05) subsequently. Lambs born to Bel and Suf × Bel ewes had a higher BW gain (*p* < 0.05) between weeks 10 and 14 than those born to Suf75. Lambs from the Suf × Bel had a higher (*p* < 0.06) BW gain between birth and weaning than lambs from Suf75, with lambs from Bel ewes being intermediate. Otherwise, first-joining age and ewe genotype did not affect (*p* > 0.05) lamb growth or carcass traits, which is consistent with the similarity in drafting pattern between the two treatments and among genotypes. 

## 4. Discussion

The objective was to evaluate the effect of age at first joining on the reproductive performance of two-tooth ewes, representing three genotypes, and on the performance of their progeny from birth to slaughter. As outlined in a previous paper [2], which presented performance at 1 year, the ewe genotypes used were chosen because they were known to differ in prolificacy. The ≥75% Suffolk was used because it represents the most common genotype in lowland flocks in Ireland, where 55% of the national lowland ewe flock is classified as Suffolk-X type [18], whilst the Belclare was included because of its high prolificacy [19].

### 4.1. Age at First Joining

The two age-at-first-joining groups were managed as one flock from approximately 16 months of age onwards. One of the main reasons often cited for not joining ewes to lamb at 1 year of age is the perception of a negative impact on BW when joining to lamb at 2 years of age. Results from previous studies [4,5,7,25,26] have shown that ewes that lambed at 1 year of age were lighter when joined to lamb at 2 years of age. In the current study, ewes that had been joined to lamb at 1 year received additional supplementation during their first pregnancy to meet requirements for pregnancy and growth. Whilst the ewes that had been joined to lamb at 1 year were lighter when joined as two tooths, they were heavier at lambing and 5 weeks post lambing. This increase in BW is also reflected in these animals having a bigger body size at 31 months of age despite being smaller at 19 months of age.

Previous studies have shown that weight at joining affects the productivity of ewes aged 1 year at lambing [2,4,27]. Keady and Hanrahan [2] used the probability of rearing ≥ 1 lamb as an overall efficiency index since this encompassed ewe fertility and litter size together with ewe and lamb mortality. In the current study, the lack of an effect of BW at joining on the probability of rearing ≥ 1 lamb was expected, as earlier studies had shown that an 8.4 kg difference in BW when joined at 19 months did not affect ovulation rate, litter size or the number of lambs reared per ewe joined [8,9]. No published information was found on the effect of weight at 7 months on the productivity when joined to lamb at 2 years. The present results indicate that an increase of 15% (~7.5 kg) in ewe BW at 7 months increases of 0.05 to 0.08 (depending on genotype) in the probability of rearing ≥ 1 when lambing at 2 years, which is ~50% of the response obtained at 1 year of age. The evidence from this study suggests that BW at 7 months has an impact on lifetime productivity that is independent of any direct effect acting through BW at 19 months since the regression coefficient for the effect of BW at 7 months was 2.5 times that expected if the impact of BW at 7 months was acting through any direct effect due to BW when joined to lamb at 2 years. This implies that the stage of development of ewe lambs when joined at 7 months has important long-term consequences for adult performance. 

The number of lambs reared per ewe joined is the key factor influencing the efficiency of prime-lamb systems of sheepmeat production [10]. Whilst the ewes in the current study lambed at 2 years of age, they reared more lambs per ewe joined than the national average for lowland sheep production systems in Ireland [28]—a result similar to those from other studies using mature ewes [29]. Birth weight impacts lamb mortality since heavy lambs are prone to dystocia whilst underweight lambs are more vulnerable to hypothermia due to their high surface area per unit BW [30,31,32]. The heavier BW at birth found in the present study for lambs born to ewes lambing for the second time is similar to results from other investigators [5,25], and is possibly related to higher food intake in mid and late pregnancy, as indicted by the dams of these lambs gaining 4.6 kg from mid-January to lambing whilst ewes that had lambed for the first time only gained 1.4 kg. The heavier BW at 5 weeks of age for lambs born to ewes lambing for the second time is consistent with a previous study [5] and is probably associated with their higher birth weight, and potentially with higher milk yield of their dams due to a heavier BW at lambing. Whilst age at first joining did not significantly affect lamb mortality, total mortality was numerically lower for lambs born to ewes joined at 7 months than for lambs from ewes first joined at 19 months. This is consistent with results from a New Zealand study [7], which showed that lamb survival was higher for 2-year-old ewes that had lambed at 1 year than for controls (not joined to lamb at 1 year) and the results from a study in the United States [6]. Ewes did not lose BW between post lambing and weaning and had attained 85 and 84% mature BW for those joined for the first and second time, respectively. The tendency for a lower survival of ewes joined to lamb for the first time at 19 months essentially reflected a higher proportion culled for mastitis and unexpected decline in body condition (5% for those joined at 7 months and 10% of those not joined at 7 months). Thomson et al. [25] reported a lower survival rate to the two-tooth stage for ewes that lambed at 1 year than that for ewes first joined to lamb at 2 years; this was attributed to a lower survival of replacements that had been born as singles. These authors [25] also reported that a greater proportion of lambs reared as singles did not conceive when joined as two tooths, and were culled, but that there was no effect of age at first lambing on the survival of replacements to the two-tooth stage if they had been born as twins or triplets. 

### 4.2. Ewe Genotype

The observed differences among genotypes with respect to litter size and number of lambs reared per ewe joined are consistent with the evidence, from an extensive comparison of Belclare and Suffolk breeds as sires of first-cross ewes [11], for a difference of approximately 0.4 between the Belclare and Suffolk breeds for both traits. The relatively small difference observed between Bel and Suf × Bel genotypes is likely attributable to heterotic effects in the latter genotype. When the results of the current study are combined with those of Keady and Hanrahan [2], Bel ewes reared an additional 0.64 lambs by 28 months of age compared with Suf75 ewes.

An efficiency index is often used to compare ewe genotypes [11]. Using the same efficiency index as previously [2] the Bel and Suf × Bel genotypes had a greater probability of rearing ≥ 1 one lamb than Suf75. The effect of genotype on this index may be attributable in part to the lower litter size of the Suf75 ewes alluded to earlier in relation to the effect of weight at 7 months. Thus, a 15% increase (~7.5 kg) in BW at 7 months had a combined impact on the average probability of rearing ≥ 1 lamb, when lambing at 1 and 2 years of age, of 0.06, 0.10 and 0.12 for the Bel, Suf × Bel and Suf75 genotypes, respectively.

Lamb output at weaning (per ewe joined), reflecting the number of lambs reared and lamb BW at weaning, is a key indicator that should inform the choice of ewe genotype used in sheepmeat systems. Keady and Hanrahan [9] found that, for a set of ewe genotypes in Ireland, the weight of lamb reared per ewe joined differed by up to 221%. Suffolk-type ewes are the dominant breed on lowland sheep farms in Ireland, accounting for 55% of ewes [18]. In the current study, relative to the Suf75 genotype, the weight of lamb reared was 26 and 28% higher for the Bel and Suf × Bel genotypes, respectively. The increased weight of lamb reared by the Bel ewes compared to Suf75 is similar to the difference reported by Keady and Hanrahan [2] for replacements joined to lamb at 1 year.

### 4.3. Lamb Performance at Pasture

Achieving a high level of lamb performance from grazed pasture is a key element in achieving high efficiency in grass-based systems of prime-lamb production [10]. The grazing management used in the present study followed the sward height profile indicated by Keady [33]. Thus, sward height post grazing was allowed to increase as the season progressed to prevent lambs from grazing the lower horizons of the sward canopy, which have lower digestibility. Ewes grazed pastures vacated by lambs during the post-weaning period in order to reduce residual sward height and thus remove some of the less digestible forage. The BW gain of lambs between birth and weaning (258 g/day) was similar to that obtained under Irish conditions by Keady and Hanrahan [9] and Earle et al. [29] using two-tooth ewes and predominantly multiparous ewes, respectively; but was less than that reported for other similar Irish studies [12,24,34,35] and by Keady et al. [36] for data over 12 consecutive seasons for a rotational-grazing system of prime-lamb production. The latter difference is attributable, at least in part, to the fact that in the present study dams were only 2 years old and that the growth rate of lambs from 2-year-old ewes is typically approximately 93% of the average for a mixed age flock (Hanrahan JP –unpublished data).

All lambs were finished prior to the end of the grazing season without any concentrate supplementation (except prior to weaning for the few lambs that were reared as triplets), demonstrating what is achievable from good grassland-management practices, as reported previously [2,9,24,33,34,35,36]. 

## 5. Conclusions

It is concluded that there is a positive relationship between weight at 7 months and the probability of rearing at least one lamb when lambing at 2 years of age and that the effect was approximately 50% of the response observed when lambing at 1 year of age. Whilst joining replacements at 7 months of age reduced BW at 19 months, these ewes were heavier at lambing and at 5 weeks post lambing than ewes first joined at 19 months. The higher BW of the ewes lambing for the second time at 2 years is probably the reason for the increased BW of their lambs at birth. Age at first lambing had no effect on ewe productivity or on progeny performance when ewes lambed at 2 years of age. Bel and Bel × Suf ewes reared more lambs per ewe joined, and thus produced more lamb carcass, than Suf75 ewes. The results also show that all lambs, except those reared as triplets preweaning, from 2-year-old ewes can be finished off pasture, prior to the end of the grazing season, without any concentrate supplementation. 

## Figures and Tables

**Table 1:** Effects of age at first joining and ewe genotype on ewe body weight and condition score, and body measurements for ewes lambing at 2 years of age.

		Age at First Joining (J)			Genotype (G)					Significance ^$^		
		7 Months	19 Months	s.e.	Belclare (B)	B × Suffolk	≥75%Suf ^†^	s.e.		J	G	J × G
BW (kg)												
	-pre joining (end Sep)	59.8	61.8	0.42	59.2 ^a^	61.1 ^b^	62.0 ^b^	0.51		***	***	ns
	-post joining (end Nov)	64.0	65.7	0.43	63.1 ^a^	65.3 ^b^	66.1 ^b^	0.53		**	***	ns
	-mid January	59.9	60.9	0.49	60.3	60.8	60.3	0.60		ns	ns	*
	-at lambing (Mar/Apr)	64.5	62.3	0.54	61.3 ^a^	64.2 ^b^	64.5 ^b^	0.66		**	***	ns
	-5 wk post lambing	65.2	63.6	0.56	63.4	64.5	65.4	0.69		*	ns	ns
	-at weaning (Jul)	64.9	64.3	0.56	64.3	64.7	64.8	0.68		ns	ns	*
	-at 31 months (Oct)	67.7	67.0	0.55	66.9	67.3	67.8	0.68		ns	ns	*
BCS												
	-pre joining	3.2	3.5	0.02	3.4	3.3	3.4	0.03		***	ns	ns
	-post joining	3.4	3.6	0.02	3.5	3.4	3.5	0.02		***	ns	ns
	-mid January	3.3	3.4	0.02	3.3	3.3	3.4	0.03		***	ns	ns
	-at lambing	3.1	3.0	0.03	3.0	3.1	3.1	0.04		ns	ns	ns
	-5 wk post lambing	3.1	3.0	0.03	3.0	3.1	3.1	0.04		ns	ns	*
	-at weaning	3.3	3.2	0.03	3.2	3.2	3.3	0.04		ns	ns	*
Survival to 31 months (%)		83	79	-	83	80	80	-		ns	ns	ns
Body measurements at 31 months (cm)												
	-withers height	67.2	67.4	0.18	68.6 ^a^	66.9 ^b^	66.4 ^b^	0.22	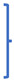	*^‡^	***^‡^	*p* < 0.07 ^‡^
	-body length	61.1 ^a^	60.4 ^b^	0.20	61.3 ^a^	60.7 ^ab^	60.2 ^b^	0.24				
	-body girth	100.9	100.8	0.38	100.6	101.3	100.6	0.47				
	-cannon bone circumference	9.4	9.4	0.04	8.8 ^a^	9.6 ^b^	9.9 ^c^	0.05				

^†^ ≥75% Suffolk ancestry. ^a,b^ Genotype means with different superscripts are significantly different at *p* < 0.05. **^$^** ns *p* > 0.05, * *p* < 0.05, ** *p* < 0.01, *** *p* < 0.001. ^‡^ Results of multivariate tests for body measurements.

**Table 2:** Effects of age at first joining and ewe genotype on litter size, number reared, lambing assistance and lamb mortality for ewes lambing at 2 years of age.

		Age at First Joining (J)			Genotype (G)				Significance ^$^		
		7 Months	19 Months	s.e.	Belclare (B)	B × Suffolk	≥75%Suf ^†^	s.e.	J	G	J × G
Litter size		1.77	1.78	0.44	1.90 ^a^	1.84 ^a^	1.59 ^b^	0.056	ns	***	ns
Number of lambs reared											
	-per ewe joined	1.41	1.38	0.052	1.50 ^a^	1.48 ^a^	1.21 ^b^	0.064	ns	**	ns
	-per ewe lambed	1.54	1.50	0.047	1.61 ^a^	1.59 ^a^	1.36 ^b^	0.058	ns	**	ns
Assisted at lambing (%)		31	33	-	31	30	36	-	ns	ns	ns
Failed to lamb (%)		9	6	-	7	5	11	-	ns	ns	*p* < 0.06 ^‡^
Lamb mortality ^§^ (%)											
	Born dead (%)	4.3	6.2	-	5.7	5.1	4.8	-	ns	ns	ns
	Total (%)	9.5	12.8	-	10.6	9.7	13.0	-	ns	ns	ns

^†^ ≥75% Suffolk ancestry. ^a,b^ Means with a different superscript from the set (a,b) are significantly different at *p* < 0.05. **^$^** ns *p* > 0.05, ** *p* < 0.01, *** *p* < 0.001. ^‡^ Treatment means for Bel, Suf and ≥75%Suf: 5.1, 12.1, 10.2 for J = 7 months: 8.4, 1.8, 11.4 J = 19 months; *p* = 0.057. ^§^ Twin-lamb basis.

**Table 3:** Effects of age at first joining and ewe genotype on growth and carcass traits of lambs born to 2-year-old ewes.

		Age at First Joining (J)			Genotype (G)				Significance ^$^		
		7 Months	19 Months	s.e.	Belclare (B)	B × Suffolk	≥75%Suf ^†^	s.e.	J	G	J × G
BW (kg)											
	-at birth	4.9	4.7	0.07	4.7	4.8	4.9	0.08	**	ns	ns
	-at weaning	30.4	30.0	0.37	30.0	30.9	29.6	0.43	ns	ns	ns
	-at slaughter	47.6	47.7	0.43	47.7	48.4	46.9	0.52	ns	ns	ns
ADG (g)											
	-birth to 5 wk	284	273	4.5	275	286	274	5.3	*p* < 0.06	ns	ns
	-5 to 10 wk	294	294	4.4	291	298	294	5.1	ns	ns	ns
	-10 to 14 wk	178	188	7.1	193 ^a^	193 ^a^	164 ^b^	8.4	ns	**	ns
	-birth to weaning	259	258	3.5	259 ^ab^	265 ^b^	252 ^a^	4.1	ns	*p* < 0.06	ns
	-birth to slaughter	226	224	2.9	225	229	222	3.4	ns	ns	ns
Carcass weight ^‡^ (kg)		20.7	20.5	0.13	20.6	20.7	20.4	0.16	ns	ns	ns
Carcass fat score ^µ^		2.8	2.9	0.04	2.9	2.8	2.8	0.04	ns	ns	ns
Dressing proportion (g/kg)		420	417	2.0	421	418	417	2.4	ns	ns	ns
Age at slaughter ^‡^ (d)		202	204	2.4	203	201	204	2.8	ns	ns	ns

^†^ ≥75% Suffolk ancestry. ^a,b^ Means with a different superscript from the set (a,b) are significantly different at *p* < 0.05. **^$^** ** *p* < 0.01. ^µ^ At carcass weight = 20 kg. ^‡^ At fat score = 3.

## Data Availability

The data reported within this study are available from the corresponding author upon reasonable request.
